# The Psychological Factor in Securing Central Occlusion

**Published:** 1922-04

**Authors:** Dayton Dunbar Campbell

**Affiliations:** Kansas City, Missouri


### THE PSYCHOLOGICAL FACTOR IN SECURING CENTRAL OCCLUSION

BY DAYTON DUNBAR CAMPBELL, D.D.S., KANSAS CITY, MISSOURI

The "Central Occlusion" desired by the prosthetist is the normal or natural occlusion of the patient. This normal or natural occlusion is easiest to obtain when the other actions of the patient are normal or natural. These actions are normal or natural or habitual when the patient's processes of thought are following accustomed or habitual channels. The problem of securing or maintaining this mental condition is the psychological problem confronting the prosthetist when he wishes the patient to close upon the occlusal rims.

The psychological factor involved in the process of securing central occlusion is so important that the writer is tempted to assert that it outweighs the physical or mechanical provisions together with the physiological considerations of the case. The writer believes that all of his hearers are agreed with him that the psychological factor in denture construction can be so vitally effective in a negative way that the most exact workmanship as well as the most favorable conditions of mouth tissue will not assure successful dentures.

In making preparations to secure central occlusion we should bear in mind that the closing movement desired is in many respects similar to other movements which, taken in series, form habits, and which, as habits, are usually performed most easily when the mind of the performer is not concentrated upon any particular movement of the series. The effort to separate an act from the series in the midst of which it usually occurs is often not only confusing but utterly unavailing. In fact, some of the most simple habits we have never analyzed; for example, in dressing—do you put your right arm into your coat and then put in your left, or do you first put in your left? In jumping a running broad jump do you ''take off'' from your right foot or your left? In starting your follow-through stroke are your elbows above the plane of your hips or below? In getting off a moving train from the left side is your left or right foot the first to touch the pavement? Is it the same when you alight from the right side? (Of course, in the case of a woman alighting from a street car it's probably wrong!)

Such instances will serve to emphasize the difficulty confronting the patient if he is asked to close ''naturally,'' or when he is told that we are now going to get ''your regular bite.'' He has probably never until that moment thought of how he has been in the habit of closing his jaws; and his confusion will be greater than that caused by the questions we have just suggested, because of the fact that it is even more unlikely that he has analyzed this movement. So, if instead of asking a singer to sound a particular note which occurs in the middle of a melody, we ask her to sing the melody and we prepare ourselves to take notice of the peculiar quality of the note as she sings; or, if instead of asking the pianist whether he starts the difficult arpeggio with little finger or thumb, we watch the fingering of the measure while the selection is being played, we shall obtain a more satisfactory reply than if we insisted upon the singer's or player's unnatural and non-habitual concentration upon a unit of the series. Likewise, if the prosthetist can take his central occlusion from a series of closing movements made by the patient, he will secure a habitual relationship of upper and lower which, incorporated in the artificial dentures, will be found to be correct.

While the factor to which we are calling attention is a mental one, it is influenced very largely by material factors which are almost wholly within the prosthetist's control. Consequently, the psychological problem is in large measure solved when the prosthetist has made careful preparations in anticipation of the final movement in the procedure.

Quite obviously, the material factors which will be most vital in securing central occlusion are the occlusal rims. These should be constructed with much care, and the finish and polish should be comparable, so far as the material permits, to the finished dentures. In constructing these, the depth of the wax is always left greater than the operator believes the case will require, since it will be easier while he is at the chair to cut them down than to build them up.

The upper is placed in position and retained without any doubt by the assistance of some one of the gum tragacanth or similar preparations available. After marking the median and lip lines, it is removed and the six anteriors of the teeth selected for the case are waxed securely in position and returned to the mouth in an alignment that is satisfying to both patient and prosthetist as well as to relatives or accompanying friends, who we insist *must*

*be present upon this occasion.* The upper is removed and the wax posterior to the cuspids is cut away, forming a wedge-shaped ridge in order to offer less resistance in the moment of closing.

The lower occlusal rim is placed in position, the lower lip line noted and then removed. The excess wax above the lip line is cut away the entire length of the rim, and in many instances it is necessary to shorten the base at the tuberosities three-eighths to a half-inch, in order to assure in the act of closing freedom from interference with the upper. The entire body of wax to be occupied by the six lower anteriors is cut away and a central incisor placed in the median line in order to maintain the lip line and also serve as a stop when the jaw closes to central occlusion. The entire surface of the wax is again smoothed.

With a sizzling hot spatula the remaining body of wax on each side of the lower is softened through to the base everywhere, except at the confining borders, until each is a veritable pool. The upper occlusal rim is now placed in position and as soon as the pools of the lower have congealed sufficiently to keep them from overflowing, the lower is inserted and held in position on the ridge, using the index finger of each hand for this purpose. The patient should be sitting erect with head free from the head-rest, or standing with face toward the light. As the operator approaches the patient with the lower ready to insert, he opens his own mouth rather wide and the patient responds to the suggestion by opening his mouth in like fashion to receive the occlusal rim. When the rim is in position and held as we have indicated, the operator closes his own mouth with a click or snap to central occlusion and the patient responds to the suggestion with a similar action.

The physical behavior following the first sense of touch which comes at the moment of practically non-resisting contact of the lower with the upper, is similar to any other habitual response to a stimulus, and having no other guide, (of course the soft wax is not a true guide such as the planes of the cusps of the teeth provide), the mandible closes in correct relationship to the maxilla. The lower central incisor, which has been plaed in or near the median line, touches the wax immediately behind the two central incisors of the upper and the closing movement is arrested with the lips in proper relationship.

In summarizing the whole viewpoint as presented, the following concrete suggestions presented in negative form may be practically helpful:

*First.* Never mention to the patient any specific movement desired, such as *correct bite,* or suggest that you want to get the *natural* or *normal* relationship of the jaws.

*Second.* Never sound a note of disapproval because of any movement that the patient may make.

*Third.* Never show by facial expression your disapproval or disappointment when movements of the patient do not contribute to success.—*Dental Summary.*

